# Intra-CA1 Administration of Minocycline Alters the Expression of Inflammation-Related Genes in Hippocampus of CCI Rats

**DOI:** 10.3389/fnmol.2019.00248

**Published:** 2019-10-24

**Authors:** Li He, Rui Xu, Yuanshou Chen, Xiaohong Liu, Youfu Pan, Song Cao, Tao Xu, Hong Tian, Junwei Zeng

**Affiliations:** ^1^Department of Physiology, Zunyi Medical University, Zunyi, China; ^2^Department of Genetics, Zunyi Medical University, Zunyi, China; ^3^Department of Pain Medicine, Affiliated Hospital of Zunyi Medical Univerisity, Zunyi, China

**Keywords:** neuropathic pain, hippocampus, microglia, toll-like receptor, chemokines

Some recent evidence suggests that microglia activation and inflammatory cytokine production in the hippocampus are associated with the development of pain behavior following peripheral nerve injury. We observed sciatic nerve chronic constriction injury (CCI)-induced inflammation-related gene expression changes that are modulated by minocycline in rat hippocampus. Intra-CA1 administration of minocycline was applied after nerve injury. Genome-wide mRNA expression in the hippocampus was evaluated to monitor the fundamental gene expression levels. We found that minocycline treatment produces a pronounced inhibition of CCI-induced mechanical allodynia. We identified 790 genes differentially expressed in CCI vs. sham rats. Among these changed genes, the 425 differentially expressed genes showed a significantly different effect in CCI vs. minocycline-treated rats. Moreover, 390 transcripts were characterized by an increase in mRNA abundance after nerve injury, and minocycline treatment reduced the level of these changes. Only 35 transcripts were characterized by a decrease in mRNA after nerve injury, and minocycline treatment reversed the decrease in the hippocampus. Noteworthily, cytokine-cytokine receptor interaction and the toll-like receptor signaling pathway are the top two most significantly enriched KEGG (kyoto encyclopedia of genes and genomes) terms in comparing the sham vs. CCI group and CCI vs. minocycline-treated group. Nine kinds of transcription factor gene transcripts (Runx3, Tfec, Pax-1, Batf3, Sp5, Hlx, Nfkbiz, Spil, Fli1) increased in abundance after nerve injury, and minocycline treatment reversed these changes. Afterwards, we selected some genes for further validation by using quantitative PCR: interleukins (Il1β), chemokines (Cxcl13, Cxcl1, Ccl2, Cxcl11, Ccl7, Ccl20), toll-like receptors (Tlr8 and Tlr1), and transcription factors (Runx3, Nfkbiz and Spil). We suggested that the transcriptional changes of these inflammation-related genes are strongly related to the processes of microglia activation underlying neuropathic pain development.

## Introduction

Neuropathic pain is a chronic pain condition that is usually induced by peripheral nerve injury. Recent reports suggest that the inflammation-related cytokines accumulation in dorsal root ganglion, dorsal spinal cord, hippocampus, thalamus, and somatosensoric cortex are paralleled by pain responses in different animal models of neuropathic pain (Al-Amin et al., 2011; Sun et al., 2016; Chang et al., 2018; Liu et al., 2018). In the chronic constriction injury (CCI) and the spared nerve injury models of neuropathic pain in rats, an increase in interleukin 1 beta (IL-1β), interleukin 6 (IL-6), nerve growth factor (NGF), and glial cell-derived neurotrophic factor (GDNF) was observed in most brain regions (Al-Amin et al., 2011). The overproduction of tumor necrosis factor-α (TNF-α) may regulate synaptic plasticity in the rat hippocampus through microglia-dependent mechanism after spared nerve injury of the sciatic nerve (Liu et al., 2017). However, it is not clear whether other inflammation-related neuroactive substances will be affected after microglia activation in the rat hippocampus after peripheral nerve injury.

It is clear that many kinds of toll-like receptors (TLRs) are expressed in the hippocampus and act as a type of pattern-recognition receptor that participate in inflammatory responses. TLR1 expression in the hippocampus was increased in the neurons, microglia, and astrocytes in seizure mice (Wang et al., 2015). TLR (2, 3, 4, 7, and 9) expression was upregulated in the hippocampus of restraint stressed rats (Timberlake et al., 2018). TLR2 and TLR4 in the rat hippocampus are related to the lipopolysaccharide (LPS)-induced neuron cell death (He et al., 2013; Henry et al., 2014). That TLR3-induces the increased expression of IL-1β in the rat hippocampus was suggested by Henry et al. (2014). TLR8, expressed in most regions of the brain, is associated with injury and neurite outgrowth (Ma et al., 2006). It is well known that TLR-dependent signaling is often associated with the overproduction and release of inflammatory cytokines in many different types of cells. However, the relationship between the changes of TLRs expression and microglia activation in hippocampus of CCI rats is not known.

Previous studies reveal that chemokine production is enhanced in some neuroimmunological diseases accompanied by pathological pain (Cartier et al., 2005). CXCL13 is obviously upregulated in the spinal cord after spinal nerve ligation and induces astrocyte activation via its receptor CXCR5 (Zhang et al., 2017). Chemokine CCL2 (C-C motif ligand 2) in the rostral ventromedial medulla is related to the descending pain facilitation in nerve-injured rats (Guo et al., 2012). Expression of chemokines CCL2 and CCL3 was increased in the thalamus and hippocampus after severe spinal cord injuries (Knerlich-Lukoschus et al., 2011). The overproduction of IL-1β and CCL2 was found in the hippocampus of CCI rats (Fiore and Austin, 2018). Moreover, Lanfranco et al. reported that CCL5 gene expression was found in neurons and glial cells in the rat hippocampus (Lanfranco et al., 2018). However, no evidence directly addresses the relationship between microglia activation and chemokine accumulation in neuropathic hypersensitivity.

It is clear that minocycline is an important modulator of the immune response and easily permeates the blood-brain barrier (Stolp et al., 2007; Vonder Haar et al., 2014). Clinically, minocycline can be administered by the intravenous route in patients with traumatic brain injury (Rojewska et al., 2014). More recent evidence suggest that minocycline is effective at reducing the spontaneous pain behavior in animal models of neuropathic pain, and that means it appears to be a promising analgesic drug (LeBlanc et al., 2011; Rojewska et al., 2014). In the present study, minocycline is applied to identify what inflammation-related genes at the hippocampus are closely related to the increased microglia activity in CCI-induced neuropathic pain rats.

## Materials and Methods

### Experimental Animals

In the experiments, adult male Sprague-Dawley (SD) rats (200–220 g) were housed under a 12: 12 h revised light/dark cycle. The protocol was prepared from SD rats in accordance with the National Institutes of Health guidelines in a manner that minimized animal suffering and animal numbers. All experiments involving animals were approved by the Zunyi Medical University Committee on Ethics in the Care and Use of Laboratory Animals.

### Intra-hippocampal Injection

Rats were anesthetized by pentobarbital sodium (40 mg/kg, i.p.) and mounted in a David Kopf stereotaxic frame (Model 1900, Tujunga, CA, USA) with a flat skull position. An incision was made along the midline and the scalp was retracted. The area surrounding the bregma was cleaned. Stainless steel guide cannulae were unilaterally implanted 1 mm above the CA1 according to rat brain atlases. Two holes were drilled through the skull and two stainless steel needles (28 gauge) were inserted through the holes (A/P-3.3 mm caudal to the bregma, L/R ± 2.0 mm lateral to the midline, D/V2.8 mm ventral to the skull surface) (Paxinos and Watson, 1998). These rats were allowed to recover for 6 days before CCI operation. A total of 0.5 μl of either PBS or minocycline was infused (0.167 μl/min, 3 min) (Zhang et al., 2016). After infusion, needles remained in place for an additional 3 min to avoid reflux. After nerve injury, the rats received bilateral intra-hippocampal treatment of 0.5 μl of either vehicle or minocycline (1, 2, 5, 10, and 15 μg/μl, twice a day) for 7 days consecutively.

### The Chronic Constriction Injury (CCI) Model

Rats were anesthetized with pentobarbital sodium (40 mg/kg, i.p.), and the sciatic nerve (left) was exposed. The left sciatic nerve was exposed and a 15-mm length of sciatic nerve proximal to the sciatic trifurcation was dissected. Four loose ligatures (4.0 braided silk) were made around the sciatic nerve at 1-mm intervals. Sham rats underwent the same procedure but without nerve ligation. After surgery, rats were housed in separate cages (at room temperature for 24 h) to avoid scratching each other (Safakhah et al., 2017; Liu et al., 2018). Rats that exhibited motor deficits such as hind-limb paralysis, impaired righting reflexes, and hind-limb dragging were excluded. That is to say, after implantation of a cannula into the hippocampus, the hind limb function of rats used for CCI and behavioral testing was not to be impaired (Huang et al., 2018). Hernández-López et al. also reported that stereotactic surgery for cannula placement in the dorsal hippocampus does not impair the motor coordination of rats (Hernández-López et al., 2017). In addition, rats not exhibiting pain hypersensitivity after nerve injury were excluded.

### Behavioral Assessment

Mechanical withdrawal threshold (MWT) was recorded to assess the response of the paw to mechanical stimulus. An electronic von Frey plantar aesthesiometer (IITC, Wood Dale, IL, USA) was used (Huang et al., 2018). After habituation to the test environment, the measurements were made. Baseline values were obtained before surgery. Mechanical stimulation was applied against the mid-plantar area of the left hind paw, and brisk withdrawal or paw flinching was considered to be positive behavior. The MWT was recorded and the cut-off force was set at 60 g. Three successive stimuli were applied, and MWT was represented by the mean values.

### Transcriptional Profile Analysis

Male SD rats were divided into Sham, CCI+0.01M PBS and CCI+ Minocycline groups (*n* = 3 per group). Three subjects from each group who met all inclusion criteria (see below) were subjected to microarray analyses. At 7 days following CCI or sham surgery, the rats were anesthetized with pentobarbital sodium (40 mg/kg, i.p.). The hippocampus of rats was dissected, flash-frozen (in liquid nitrogen) and stored at −80°C for analysis. According to the procedures described in the manual, total RNA was isolated from hippocampal tissue using TRI Reagent (Sigma Aldrich, USA). RNA degradation and contamination were checked by gel electrophoresis. The quantity of each RNA sample obtained was checked using the NanoPhotometer® spectrophotometer (IMPLEN, CA, USA) with pass criteria of absorbance ratios of A260/A280 ≥ 1.8 and A260/A230 ≥ 1.6. RNA concentrations were assessed using Qubit® RNA Assay Kit in Qubit® 2.0 Fluorometer (Life Technologies, CA, USA). A total amount of 2 μg RNA per sample was used to construct the cDNA library.

First-strand cDNA was synthesized using a HiFiScript gDNA Removal cDNA Synthesis Kit (CWBIO, Beijing, China) according to the standard protocols. Quantitative real-time PCR was carried out using a QuantStudio™ 6 Flex Real-Time System (Applied Biosystems, USA) with UltraSYBR Mixture (CWBIO, Beijing, China). The following PCR amplification program was used: 95°C for 2 min, followed by 40 cycles of 95°C for 10 s, 50–54°C (changed according to the primer sequences) for 20 s and 72°C for 20 s. A dissociation curve was performed (55–95°C) after the last PCR cycle to ascertain the specificity of the amplification reactions. The abundance of each mRNA was normalized with respect to the endogenous housekeeping gene β-actin, and the relative gene expression levels were determined by the ^2−ΔΔ^Ct method.

Microarray experiments were performed to determine gene-expression profiles in rat hippocampus. Based on the differentially expressed gene (DEG) results, the heat maps were constructed using Multiexperiment Viewer (MeV; http://mev.tm4.org/). Gene ontology (GO) and pathway enrichment analyses were carried out with the aid of the NCBI COG (http://www.ncbi.nlm.nih.gov/COG/), Gene Ontology Database (http://www.geneontology.org/) and KEGG pathway database (http://www.genome.jp/kegg/).

The DEGs were ascertained using the DESeq R package (1.10.1) as detailed in a previous study (Wang et al., 2010). False discovery rate (FDR) was used to correct the results for *P*-value. FDR ≤ 0.05 and an absolute value of log2 (fold-change) ≥1 were used as the threshold for screening DEGs. Pathway functional enrichment analysis was performed using the “phyper” function in R. The *P*-value calculating formula is:

$$\begin{array}{l} P=1-\overset{\text{m}-1}{\underset{i=0}{\sum }}\frac{\left(\begin{array}{c} \text{M}\\ \text{i} \end{array}\right)\left(\begin{array}{c} \text{N-M}\\ \text{n-i} \end{array}\right)}{\left(\begin{array}{c} \text{N}\\ \text{n} \end{array}\right)} \end{array}$$

Here, M is the number of genes in the pathway, N is the total number of genes in the genome, m is the number of target gene candidates in M and n is the number of differentially expressed genes. In addition, i = 1, 2, 3, … (M-1) where M represents the number of genes in the pathway. The Fisher's score indicates the ratio of genes (number m) belonging to the functional pathway out of the total differentially expressed genes (number n) (Zhang et al., 2018). Subsequently we calculate the value of FDR. FDR ≤ 0.01 is considered as significantly enriched.

### RT-PCR

Male SD rats were divided into Sham, Sham+Minocycline, CCI+0.01M PBS and CCI+ Minocycline groups (*n* = 6 per group). The changes in the abundance of some gene transcripts in the rat hippocampus after nerve injury and the modulatory effects of minocycline should be further investigated by PCR analysis of samples independent from those used for the microarray studies. According to the methods mentioned above, four groups of animals were treated and killed by cutting their necks. Brain tissue was quickly dissected on the ice platform and was immersed and washed with phosphate buffered solution (PBS). The hippocampus was isolated and rapidly transferred into separate RNase-free 1.5 ml Eppendorf tubes. Total RNA was immediately isolated using the TRIzol Reagent (MRC Co., Cincinnati, USA). The concentration and purity of RNA samples were measured using Spectrophotometer (Thermo Fisher Scientific). The ratios of OD260/OD280 were between 1.9 and 2.1. cDNA was synthesized from RNA by reverse transcription reaction using the SuperScript II reverse transcriptase kit (Invitrogen). All primers are shown in Table 1. qPCR was performed in a final volume of 20 μl (8 μl H_2_O, 10 μl mastermix, 1 μl assay-mix, and 1 μl cDNA) on a Linegene Real-time PCR detection system (Bioer Technology, China). PCR reaction conditions were as follows: (1) 95°C 8 min 1 Cycle; (2) 95°C 15 s and 60°C 1 min, 40 Cycles. The experimental data analysis was carried out using the ^2−ΔΔ^Ct method (Livak and Schmittgen, 2001).

**Table 1:** Primers used for RT-PCR.

**Gene**	**Forward**	**Reverse**
Cxcl13 (NM-001017496.1)	5′-TTTGGTAACCATCTGGCAGTA-3′	5′-GCTCGACCTTTATCAATCTAAT-3′
Cxcl1 (NM-030845.1)	5′-TGGCTATGACTTCGGTTTGGGT-3′	5′-GGCAGGGATTCACTTCAAGAACA-3′
Ccl2 (NM-031530.1)	5′-GTGCTGAAGTCCTTAGGGTTG-3′	5′-GTCGGCTGGAGAACTACAAGA-3′
Cxcl11 (NM-182952.2)	5′-CCAGGCACCTTTGTCCTTTAT-3′	5′-GGTTCCAGGCTTCGTTATGTT-3′
Ccl7 (NM-001007612.1)	5′-CACCGACTACTGGTGATCTTTC-3′	5′-TTCATCCACTTGCTGCTATGT-3′
Ccl20 (NM-019233.1)	5′-GACAAGACCACTGGGACA-3′	5′-AGCCTAAGAACCAAGAAG-3′
Iba-1 (NM-017196.3)	5′-CAAGGATTTGCAGGGAGGA-3′	5′-CAGCATTCGCTTCAAGGACATA-3′
Cd68 (NM-001031638.1)	5′-TCAAACAGGACCGACATCAGA-3′	5′- ATTGCTGGAGAAAGAACTATGCT-3′
iNOS (NM-012611.3)	5′-GATGTGCTGCCTCTGGTCCT-3′	5′-GAGCTCCTGGAACCACTCGT-3′
IL-1β (NM-031512.2)	5′-CAGCCTTACTGGCCTGCTAC-3′	5′-CTGCTACCACGACAGCCATA-3′
Tlr8 (NM-001101009.1)	5′-TGCTTCATTTGGGATTTG-3′	5′-TGGCATTTACACGCTCAC-3′
Tlr1 (NM-001172120.2)	5′-CAGTTTCTGGGATTGAGCGGT-3′	5′-TAATGTGCTGAAGACACTTGGGATC-3′
Runx3 (NM-130425.1)	5′-GGCTTTGGTCTGGTCCTCTATC-3′	5′-GCAACGCTTCCGCTGTCA-3′
Nfkbiz (NM-001107095.1)	5′-CCGTAGAAGTAAGCGAGGTT-3′	5′-GAGCATGATCGTGGACAAG-3′
Spil (NM-001005892.2)	5′-CAATCTTTGCTCCTCTTT-3′	5′-CTACCAATCCTGGCTTCA-3′
β-actin (NM-031144.3)	5′-AGCCATGTACGTAGCCATCC-3′	5′-ACCCTCATAGATGGGCACAG-3′

### Statistical Analysis

All data were presented as mean ± standard deviation (SD.). The behavioral and PCR data were analyzed by one- (compared within the group) or two-way (compared between groups) ANOVA. If significance was established, *post-hoc* Dunnett or Bonferroni's multiple comparisons were performed. All statistical tests were carried out using SPSS 18.0 software (IBM, Armonk, NY). The level of significance was set as *p* < 0.05.

## Results

### Intra-CA1 Administration of Minocycline Attenuates CCI-Induced Mechanical Allodynia

**Figure 1:**
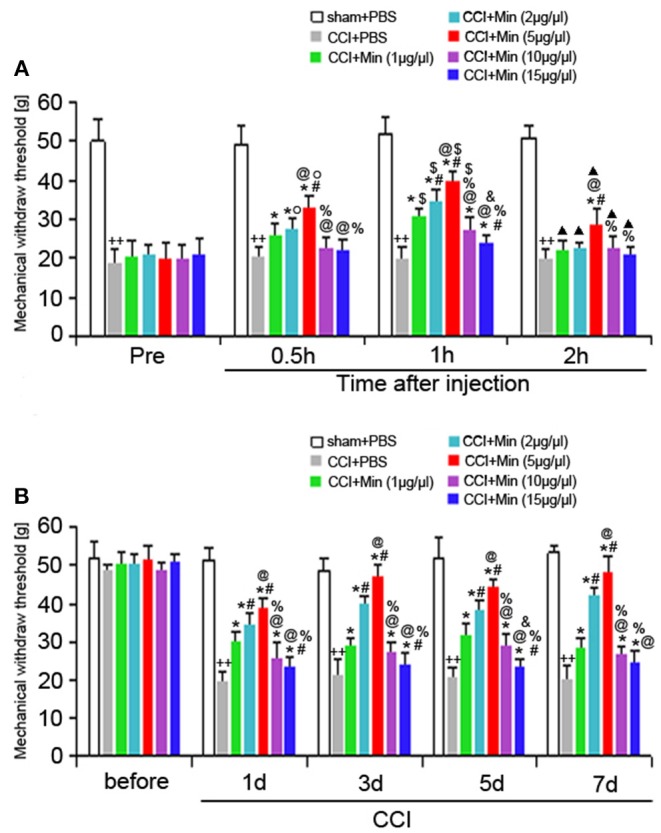
MWT was determined in different groups. All values represent mean ± SD (*n* = 8). **(A)** Decreased MWT was exhibited in the CCI rats on day 1 after surgery compared to sham rats (^++^*P* < 0.01). Compared with CCI rats, minocycline (1, 2, 5, 10, and 15 μg/μl) treatment exerted anti-hyperalgesic effects (^*^P < 0.05). Minocycline treatment showed obvious increased MWT (vs. 1 μg/μl: ^#^*P* < 0.05; vs. 2 μg/μl: ^@^*P* < 0.05; vs. 5 μg/μl: ^%^*P* < 0.05; vs. 10 μg/μl: ^&^*P* < 0.05). Greater analgesic effect of minocycline occurs 1 h after its administration (vs. Pre: ^O^*P* < 0.05; vs. 30 min: ^$^*P* < 0.05; vs. 1 h: ^▴^*P* < 0.05); **(B)** The MWT was measured at 1, 3, 5, and 7 days after surgery. Decreased MWT was exhibited in the CCI rats on days 1, 3, 5, and 7 after nerve injury compared to sham rats (^++^*P* < 0.01). Compared with CCI rats, minocycline (1, 2, 5, 10, and 15 μg/μl) treatment exerted anti-hyperalgesic effects (^*^P < 0.05). Minocycline treatment showed the more obvious increase in MWT (vs. 1 μg/μl: ^#^*P* < 0.05; vs. 2 μg/μl: ^@^*P* < 0.05; vs. 5 μg/μl: ^%^*P* < 0.05; vs. 10 μg/μl: ^&^*P* < 0.05).

To investigate the antinociceptive effect of minocycline on the mechanical nociceptive threshold in neuropathic pain rats, the MWT was recorded on the day before and after surgery (at POD 1, 3, 5, and 7). A total of five doses (1, 2, 5, 10, and 15 μg/μl, twice a day) were administered. We compared the changes of MWT between the different time points (Figure 1). Application of minocycline at 1, 2, and 5 μg/μl for 30 min showed increased MWT in comparison to CCI rats (*P* < 0.05). Application of minocycline at 1, 2, and 5 μg/μl for 1 h also showed more significant increase in MWT (vs. CCI rats: *P* < 0.01; vs. 30 min: *P* < 0.05). Application of minocycline at 10 and 15 μg/μl for 30 min showed a slight increase but was not significantly different from that of the vehicle-treated CCI group. Application of minocycline at 10 μg/μl for 1 h showed obvious increased MWT (vs. CCI rats: *P* < 0.05; vs. 30 min: *P* < 0.05). Application of minocycline at 15 μg/μl for 1 h also showed increased MWT (vs. CCI rats: *P* < 0.05). Application of minocycline at 5 μg/μl for 2 h showed slightly increased MWT (vs. CCI rats: *P* < 0.05; vs. 1 h: *P* < 0.01). These results suggest that minocycline produced a reversal of MWT, with maximal effect at 1 h after minocycline administration.

As shown in Figure 1B, decreased MWT was observed in rats on day 1 after surgery compared to sham rats (*P* < 0.05), and the allodynia was sustained throughout the experimental period. Compared to the vehicle-treated CCI rats, minocycline at 1 μg/μl induced significant analgesic effect (*P* < 0.05). Minocycline at doses of 2 and 5 μg/μl showed better analgesic effects in comparison with minocycline at dose 1 μg/μl (*P* < 0.05). We also noticed that minocycline at a dose of 5 μg/μl showed apparent elevations of the mechanical pain threshold in comparison with minocycline at a dose of 2 μg/μl (*P* < 0.05). On the other hand, minocycline at 10 μg/μl produced moderate antinociceptive effect in CCI rats. Minocycline at 15 μg/μl induced a slight but significant antinociceptive effect in CCI rats. Minocycline at a dose of 5 μg/μl showed better analgesic effects in comparison with minocycline at doses of 10 and 15 μg/μl (*P* < 0.05). In a short, three main conclusions can be drawn: (1) The decreased MWT in CCI rats and the analgesic effect of minocycline in minocycline-treated CCI rats are maintained over 7 days; (2) a greater analgesic effect of minocycline occurs 1 h after its administration; (3) the highest analgesic effect of minocycline occurs at a dose of 5 μg/μl. Then, the minimum dose of minocycline (5 μg/μl) showing maximum effect was selected in the following experiments.

### Identification of Differentially Expressed Genes Between Different Groups

**Figure 2:**
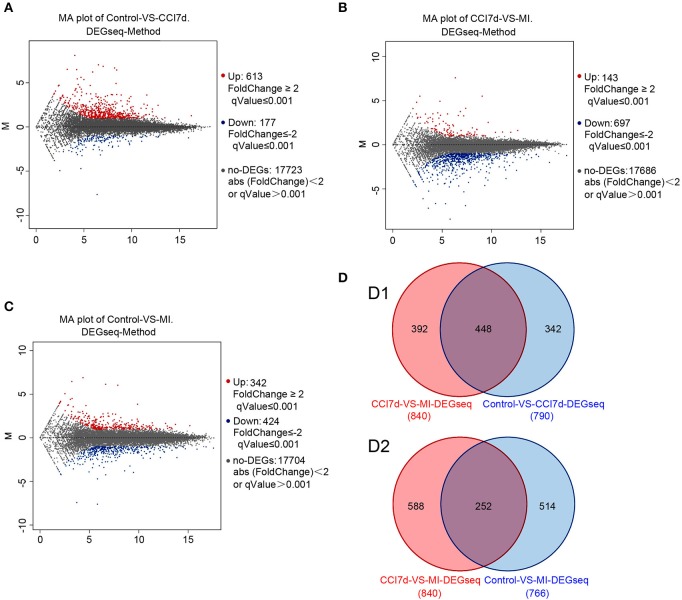
The DEGs were identified. In **(A–C)**, red dots represent increased DEGs and blue dots represent decreased DEGs. In addition, gray dots represent non-DEGs. **(A)** MA plot for DEG analysis between sham and CCI groups. **(B)** MA plot of DEGs among the hippocampus between the CCI and minocycline-treated group. **(C)** MA plot of DEGs among the hippocampus between sham and minocycline-treated group. **(D)** Comparisons of the number and overlapping DEGs between different experimental groups (the Venn diagram of DEGs). (D1) Blue circle represents number of DEGs between sham and CCI group; red circle represents number of DEGs between CCI and minocycline-treated group; the overlapping area represents shared DEGs of two comparable groups. (D2) Blue circle represents number of DEGs between sham group and minocycline-treated group; red circle represents number of DEGs between CCI group and minocycline-treated group; the overlapping area represents shared DEGs of two comparable groups.

To explore the possible role of microglia activation and inflammation within the hippocampus in the development of peripheral neuropathic pain, the DEGs between different groups were identified. According to the results, in the rat hippocampus, there were 790 DEGs between the sham group and the CCI group. Among them, 613 genes were increased and 177 were decreased (as shown in Figure 2A and Table S1). There were 840 DEGs between the CCI group and minocycline-treated group, among them 143 genes were increased and 697 were decreased (as shown in Figure 2B and Table S2). Between the sham group vs. CCI group and minocycline-treated group vs. CCI group, 448 DEGs were shared (as shown in Figure 2D and Table S3). Among these 448 DEGs, 398 transcripts were characterized by an increase in mRNA abundance after nerve injury, and minocycline application decreased the level of these changes. Only 34 transcripts were characterized by a decrease in mRNA after nerve injury, and minocycline treatment reversed the decrease in hippocampus of CCI rats (as shown in Table S3). It seems that these 432 genes may be associated with the effect of minocycline in CCI rats. In addition, only two transcripts were upregulated in CCI and upregulated by minocycline. Fourteen transcripts were downregulated in CCI and downregulated by minocycline. We also found that there were 766 DEGs between the sham group and the minocycline-treated group. Among them 342 genes were increased and 424 were decreased (as shown in Figure 2C and Table S4). Between the sham group vs. CCI group and sham group vs. minocycline-treated group, 252 DEGs were shared, among them 86 genes were increased and 166 were decreased (as shown in Figure 2D and Table S5).

### Differential Expression Analysis at the Gene Ontology Annotation Level

**Figure 3:**
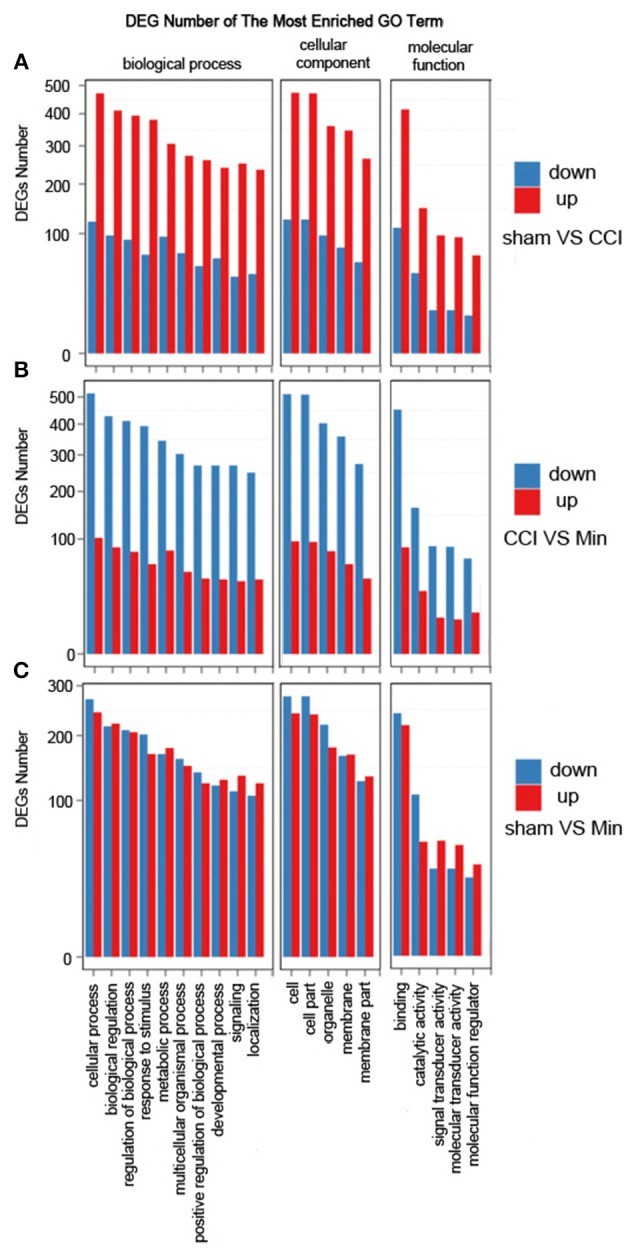
GO term classification of increased and decreased genes on DEGs for each pairwise. X axis represents GO term. Y axis represents the number of increased/decreased genes. **(A)** The most enriched GO terms between the sham and CCI groups. **(B)** The most enriched GO terms between the CCI and minocycline-treated groups. **(C)** The most enriched GO terms between the sham and minocycline-treated groups.

The DEGs were annotated covering molecular biological function, cellular component and biological process. As shown in Figure 3, the DEGs in the sham, CCI and minocycline-treated groups can be mostly classified into biological processes. The five most enriched GO terms of the DEGs for biological process were the cellular process, biological regulation, regulation of biological process, response to stimulus, and metabolic process. The five most enriched GO terms of the DEGs for the cellular component were cell, cell part, organelle, membrane, and membrane part. The five most enriched GO terms of the DEGs for molecular function were binding, catalytic activity, signal transducer activity and molecular function regulator. Compared with the sham group, the differentially expressed annotated genes in biological process, molecular function, and cellular component were mainly increased in CCI rats (Figure 3A). As far as the minocycline-treated and CCI groups were concerned, the differentially expressed annotated genes in biological process, molecular function, and cellular component were mainly decreased in the minocycline-treated group (Figure 3B). As a result, as shown in Figure 3C, between sham and minocycline-treated group, the numbers of DEGs in biological process, molecular function, and cellular component are decreased.

### KEGG Pathway Analysis of Differentially Expressed Genes

Compared with the sham-operated group, the CCI group had 20 differential gene-involved significant pathways. DEGs contained in these pathways (top 14) are shown in Table 2. Some pathogenic microorganism infection-related pathways (herpes simplex infection, tuberculosis, influenza A, malaria, Pertussis, and Leishmaniasis infection) were also involved in the process. As far as the sham and CCI groups were concerned, the most enriched KEGG pathways were the cytokine-cytokine receptor interaction pathway and the TLR signaling pathway. The cytokine-cytokine receptor interaction pathway was significantly affected, with 31 increased genes and 1 decreased gene involved in the hippocampus of CCI rats. The TLR signaling pathway was significantly affected, with 17 increased genes and 2 decreased genes involved in the hippocampus of CCI rats.

**Table 2:** The top 14 most significant KEGG pathways identified with increased and decreased genes among different groups.

**Pathway (FDR ≤ 0.01)**	**CCI group vs. sham group**	**Minocycline-treated group vs. CCI group**
Cytokine-cytokine receptor interaction	**Increased:** Cxcl11, Cxcl13, Cxcl16, Cxcl4, Ccl2, Ccl3, Ccl7, Ccl5, Ccr2, Ccr5, Osmr, Csf3, Csf3r, Il4r, Il13ra1, Csf2rb, Il2ra, Il2rb, Il2rg, Csf1r, Il10rb, IL20rb, Sf1b, Sf1a, Sf14, Fas, Cd40, Tgfβ1, Il1β, Il1r1, Il18r, Ap **Decreased:** Tnfsf15	**Decreased:** Il8ra, Cxcr3, Cxcl11, Cxcl13, Cxcl16, Cxcl4, Ccr7, Ccl2, Ccl3, Ccl5, Ccl7, Osmr, Bsf3, Csf3, Csf2rb, Il2rb, Il2ra, Il2rg, Il4r, Il21r, Il10ra, Il20rb, Ltb, Sf1b, Sf1a, Sf14, Fas, Sf9, Tnfsf4, Tnfsf13b, Il-1b, Il1r1, Il18rap
Toll-like receptor signaling pathway	**Increased:** Tlr1, Tlr2, Tlr6, Md2, P13k, Tlr7. Tlr9, Trif, Opn, Tp12, Il1β, Rantes, Mip1α, Cd40, Cd86, Itac **Decreased:** Mkk3,Irf7	**Increased:** Mkk3, Mkk6 **Decreased:** Lbp, Tlr1, Tlr2, Tlr6, Cd14, Md-2, Tlr7, Tlr8, Tlr9, Tab1, Iκbα, Il-1β, Ccl5, Mip-1α, Cd86, I-Tac
Phagosome	**Increased:** MhcI, MhcII, Fcyr, Ic3b, collectins, Tlr2, Cd14, Tlr6, Mr, Dectin1, Sra1, Tuba, Tubb, Cyba, Nox1, Ncf1, Ncf2, Ncf4, **Decreased:** Tap, Stx7	**Increased:** Stx7 **Decreased:** MhcI, MhcII, Tuba, Tubb, M6pr, Fcyr, C3, Tsp, Tlr2, Tlr6, Cd14, Mr, Dectin1, Sra1, P22phox, Gp91, P67phox, P40phox
Fc gamma R-mediated Phagocytosis	**Increased:** Fcgr2b, Cd45, Src, Lat, Pi3k, Fcyri, Fcgr2a, Plcy, Sphk, Cpkc, Ncf1, Wasp, Arpc5, Vav, Rac, Dock2, Pag3 **Decreased:** Crk	**Increased:** CrkII **Decreased:** Fcgr2b, Cd45, Fcyri, FcyrIIa, Src,Pld, Vav, CrkII, Sphk,Rac, Pag3, Wasp, Arp2, Arp3, Gsn
TNF signaling pathway	**Increased:** Tnfr1, Ciap1/2, Tp12, Rip3, Mlkl, Ccl2, Ccl5, Cxcl1, Cxcl2, Cxcl3, Fas, Il1β, Bcl3, Socs3, Ifi47, Tnfr2 **Decreased:** Mkk3	**Increased:** Mkk3, Mkk6 **Decreased:** Tnfr1, Ciap1, Ciap2, Tab1, Tab2, Tab3, Iκba, C/ebpb, Rip3, Mlkl, Ccl2, Ccl5, Cxc11, Cxc12, Cxc13, Fas, Il-1β, Bcl3, Nfkbia, Socs3, IfI47, Icam1, Tnfr2,
Complement and coagulation cascades	**Increased:** Tfpi, F10, Vwf, Par3, A2m, Pai, C3, Fd, Fb, C1qrs, C1inh, Mbl, C2, C4, C6, Cr4, C5ar1 **Decreased:** Klkb1, Tfpi, Fh	**Increased:** F5, Fh, A2m, Fga **Decreased:** F10, Par3, Par4, A2m, Pai, Upar, Bdkrb1, Bdkrb2, Cfi, Cfb, Cfd, C3, C6, C7, C8,C9, C1qa, C1inh C2, C4, C3ar1, Cr4, C5ar1
Cell adhesion molecules	**Increased:** Cd86, MhcII, MhcI, Pvrl2, Cd40, Itgal, Cd2, Cd4, Cd8, Cd6, Ptprc, Selp, Sell, Sdc, Pvrl1 **Decreased:** Cntnap2, Mpz, Mhc1	**Increased:** Cldn, Cdh2, Cdh41 **Decreased:** Cd2, Cd86, Icos, MhcII, MhcI, Cd8, Cd6. Itgal, Icam3, PtprC, Selp, Icam1, Icam2, Ngl1, Sdc, Mag
Natural killer cell mediated cytotoxicity	**Increased:** Bid, Fas, Trailr, Itgal, Shp1, Dap12, Fcer1y, FcyrIII, Nkp30, Lat, Vav, Rac, Pi3k, Plcy, Pkc	**Increased:** Rae-1 **Decreased:** Icam1, Icam2, Trailr, Fas, Itgal, FcyrIII, Shp-1, Dap-12, Fcer1g, Sap, Vav, Rac
NF-κB signaling pathway	**Increased:** Il1β, Il1r, Lyn, Lat, Btk, Plcy2, Cd14, Md2, Cd40, Trif, Ciap1/2, Carma, Bcl2a1, Baff	**Decreased:** Il1β, Il-1r, Tnf-r1, Lbp, Cd14, Md-2, Ltb, Baff, Btk, Ciap1, Ciap2, Tab, Carma, Iκbα, Il1β, Icam
Chemokine signaling pathway	**Increased:** Ac, Chemokine, Cxcr2, Gnb1, Src, Pi3k, Dock2, Rac, Vav, Wasp, Ncf1 **Decreased:** Ac, Gnb1, Crk	**Increased:** Gnb1, Crk **Decreased:** Gro, Cxcr2, Gnb1, Src, Vav, Crk,Rac, Wasp, Iκb
Osteoclast differentiation	**Increased:** Ii-1, Tgfb, Il-1r, Tnfr1, Oscar, Fcry, Dap12, Btk, Socs1, Plcy, Pi3k, Nadph, Spi1	**Decreased:** Ii-1, Il-1r, Tnfr1, Oscar, Fcry, Dap12, Btk, Socs1, Socs3, Tab1, Tab2, Nadph, Iκb, SpI1
B cell receptor signaling pathway	**Increased:** Cd72, Shp1, Lyn, Btk, FcgrIIb, Leu13, Vav, Plc-y2, Calma, Rac, Pi3k **Decreased:** Bam32	**Decreased:** Iga, Cd72, Shp1, Fcgr2b, Leu13, Btk, Vav, Rac, Card11, Iκb
Primary immunodeficiency	**Increased:** Yc, Btk, Cd45, Cd4, Cd8, Cd8α, CIIta, Cd40 **Decreased:** Tap2,Rfxap	**Decreased:** Cd3e, Il2rg, Iga, Btk, Cd45, Cd4, Cd8, Rfxap, CIIta, Cd8a, Rfxap, CIIta, Icos
Platelet activation	**Increased:** Collagen, Vwf, Par1, Fcry, Lyn, Pi3k, Btk, Plcy2, Ac, Kind3, Fcgr2a, Tbxas **Decreased:** Ac,Collagen	**Increased:** Col1a,Fg **Decreased:** Cll1a, Par1, Par4, P2x1, Fcry, Pi3k, Btk, Tbxas1, Fermt3, Fcgr2a

We also noticed that the minocycline-treated group had 20 differential gene-involved significant pathways in comparison with the CCI group. DEGs contained in these pathways (top 14) are also shown in Table 2. These results reveal that minocycline administration can regulate the expression of genes in these pathways, and reversing the gene expression changes in these pathways may be considered as one of the important mechanisms of minocycline against CCI-induced neuropathic pain. Some pathogenic microorganism infection-related pathways were also significantly downregulated. As far as the CCI and minocycline-treated groups were concerned, the most enriched KEGG pathways were the cytokine-cytokine receptor interaction pathway and the TLR signaling pathway. Among them, the cytokine-cytokine receptor interaction pathway was obviously affected, with 33 decreased genes involved. The TLR signaling pathway was significantly affected, with 16 decreased genes and 2 increased genes involved in the hippocampus of minocycline-treated CCI rats.

### mRNA Expression Profile of Inflammation-Related Genes in Rat Hippocampus

Cytokines and chemokines were originally identified as essential mediators for inflammatory and immune responses in the formation of neuropathic pain (White and Wilson, 2008; Totsch and Sorge, 2017). As shown in Table 3, in the rat hippocampus, higher transcript levels of Cxcl13, Cxcl1, Ccl2, Cxcl11, Ccl7, Ccl20, Ccl3, Ccl6, Ccl5, and Cxcl16 (Top 10 upregulated chemokine genes) were found in the CCI group as compared with sham rats. Except for Cxcl1, this upregulation of chemokine genes was almost diminished after repeated treatment with minocycline. We noticed that pro-inflammatory biomarker Il1β and iNOS are robustly upregulated at the transcriptional level after nerve injury. After minocycline treatment, iNOS and Il1β were obviously downregulated as compared with CCI rats. Il-18rap (rCG22315) transcript was also upregulated in the hippocampus, and minocycline suppressed upregulation in CCI rats.

**Table 3:** mRNA expression profile of inflammation-related genes among different groups.

**Gene symbol**	**Sham vs. CCI**		**CCI vs. minocycline**		**Sham vs. minocycline**	
	**Fold** **change**	**FDR**	**Fold** **change**	**FDR**	**Fold** **change**	**FDR**
Cxcl13	+7.03	7.02E-236	−8.42	2.75 E-207	−1.39	0.13
Cxcl1	+5.44	2.07E-31	−1.87	1.75 E-13	+3.57	2.18E-08
Ccl2	+5.35	1.35E-48	−6.65	1.77 E-47	−1.30	0.26
Cxcl11	+4.80	2.95E-26	−4.45	2.75 E-25	+0.35	0.72
Ccl7	+4.27	1.02E-17	−7.25	4.73E-18	0	0
Ccl20	+3.46	0.003003	−3.44	0.0029	0.02	0.99
Ccl3	+2.40	0.000603	−3.38	4.47 E-05	−0.98	0.42
Ccl6	+2.22	3.47E-12	−3.20	2.39 E-17	−0.98	0.07
Ccl5	+1.61	3.03E-07	−3.48	6.45 E-16	−1.87	0
Cxcl16	+1.27	6.02E-31	−1.88	1.70 E-53	−0.61	5.23E-05
INos	+5.46	2.72E-06	−5.44	2.67 E-06	0	0
Il1β	+4.34	9.98E-19	−3.15	4.47E-15	+1.19	0.15
Il18rap	+4.13	6.00 E-16	−6.11	2.35 E-17	−1.98	0.17
Socs3	+3.67	2.94 E-243	−3.08	5.23E-209	+0.58	0
C3	+3.61	0	−3.83	0	−0.22	0
Tlr8	+3.49	1.05E-11	−4.20	4.72 E-13	−0.71	0.49
Ptges	+3.47	4.28E-49	−2.48	2.02 E-35	+0.99	0
Mt1	+2.15	4.94E-228	−1.81	5.21E-181	+0.34	0
Il20rb	+2.08	1.32 E-18	−1.11	3.61 E-08	+0.97	0
Tlr1	+1.93	1.91E-09	−2.12	1.57 E-10	−0.18	0.66
Il21r	+1.92	1.10E-18	−2.01	1.21 E-19	−0.09	0.77
Il2rb	+1.69	2.99 E-06	−1.78	1.14 E-16	−0.10	0.84
Tnfrsf1b	+1.66	5.44E-32	−1.20	6.16 E-20	+0.46	0.01
Hpgds	+1.64	1.35E-07	−1.56	3.59 E-07	+0.08	0.84
Tlr13	+1.61	8.94E-20	−1.37	1.03 E-15	+0.24	0.27
I11r1	+1.48	8.55E-62	−1.15	6.06 E-42	+0.32	0
Irf8	+1.57	7.14E-85	−1.35	5.28 E-27	+0.23	0.03
Card11	+1.49	1.38E-28	−1.65	9.27 E-33	−0.16	0.36
P2ry6	+1.49	4.35E-44	−1.33	8.20 E-37	+0.17	0.21
Tlr7	+1.48	7.32E-34	−1.15	4.02 E-23	+0.33	0.02
Casp4	+1.38	3.52E-14	−1.34	1.52 E-13	+0.04	0.86
Fas	+1.22	1.79E-05	−1.37	2.68 E-06	−0.15	0.68
Tlr2	+1.06	1.61E-19	−1.05	3.82 E-19	+0.10	0.94
Tlr9	+1.06	0.0001	−1.65	8.08 E-08	−0.60	0.10
Tifab	+1.01	8.91 E-29	−1.05	4.44 E-30	−0.03	0.76
Cd68	+3.45	9.91E-95	−2.90	1.19E-80	+0.54	0.05
Msr-1	+2.01	7.93E-21	−1.94	7.27 E-20	0.07	0.82
Iba-1	+1.16	3.94E-46	−1.16	1.28E-45	0	0.98
Ox-42 (Cd11b)	+0.72	9.34E-30	−0.55	7.70E-19	+0.17	0.01
Ptges	+3.47	4.28E-49	−2.48	2.02 E-35	+0.99	0.01
Mrc1	+2.85	4.43 E-129	−2.71	6.94 E-122	+0.13	0.48
Cd86	+1.46	1.41E-07	−1.86	3.20 E-10	−0.41	0.27
Tgfβ1	+1.16	2.58 E-49	−0.96	4.06E-36	+0.20	0.03
Arg1	−1.06	8.19 E-17	+0.43	0	−0.62	5.18E-08
IL4r	+1.03	5.18 E-70	−0.14	0	+0.90	3.18E-51
Runx3	+6.39	7.42 E-11	−3.79	1.51 E-09	0	0
Tfec	+3.95	5.47 E-17	−5.25	7.13 E-19	−1.30	0.26
Pax-1	+3.70	0.0009	−4.68	0.0004	0	0
Batf3	+2.67	0.0005	−1.03	4.69 E-08	−0.11	0.62
Sp5	+2.17	6.15 E-06	−1.42	0.00076	+0.76	0.20
Hlx	+1.58	9.94 E-28	−1.26	1.85 E-11	+0.32	0.18
Nfkbiz	+1.46	9.25E-28	−2.02	1.12 E-42	−0.56	0
Spi1 (Pu.1)	+1.33	2.57E-34	−1.19	1.12 E-28	+0.15	0.27
Fli1	+1.21	3.04 E-35	−1.11	1.33 E-30	+0.10	0.39
Lst1	+2.39	0.0001	−1.29	0.01	+1.10	0.14
Maff	+1.67	1.72 E-09	−0.45	0.04	+1.23	2.50E-05
Elf4	+1.39	6.46 E-11	−0.95	1.93E-06	+0.45	0.07
Vgl13	+1.38	3.40 E-51	+0.66	2.52E-24	+2.04	2.25E-140
Nr2f2	+1.12	2.89 E-144	+0.59	1.82E-71	+1.72	0
Cartpt	−2.30	2.14 E-70	+0.17	0.23	−2.13	0
Six3	−1.68	6.84 E-06	−0.57	0.20	−2.25	1.48E-08
Meox1	−1.64	7.13 E-05	+1.11	0.01	−0.53	0.10
Tfap-2c	−1.55	1.84 E-05	+0.28	0.31	−1.27	0
Ebf3	−1.40	2.05 E-05	−0.44	0.22	−1.84	7.32E-08
Mkx	−1.12	2.27 E-08	−0.003	0.49	−1.12	7.17E-09
Mei4	−1.00	2.91 E-05	+0.09	0.4	−0.91	6.09E-05

*Cxcl13, C-X-C motif chemokine ligand 13; Cxcl1, C-X-C motif chemokine ligand 1; Ccl2, C-C motif chemokine ligand 2; Cxcl11, C-X-C motif chemokine ligand 11; Ccl7, C-C motif chemokine ligand 7; Ccl20, C-C motif chemokine ligand 20; Ccl3, C-C motif chemokine ligand 3; Ccl6, C-C motif chemokine ligand 6; Ccl5, C-C motif chemokine ligand 5; Cxcl16, C-X-C motif chemokine ligand 16; Nos, nitric oxide synthase 2; Il1β, interleukin 1 beta; Il18rap, interleukin 18 receptor accessory protein; Socs3, suppressor of cytokine signaling 3; C3, complement C3; Tlr8, toll-like receptor 8; Ptges, prostaglandin E synthase; Mt1, metallothionein 1; Il20rb, interleukin 20 receptor subunit beta; Tlr1, toll-like receptor 1; Il21r, interleukin 21 receptor; Il2rb, interleukin 2 receptor subunit beta; Tnfrsf1b, TNF receptor superfamily member 1B; Hpgds, hematopoietic prostaglandin D synthase; Tlr13, toll-like receptor 13; Il1r1, interleukin 1 receptor type 1; Irf8, interferon regulatory factor 8; Card11, caspase recruitment domain family, member 11; P2ry6, pyrimidinergic receptor P2Y6; Tlr7, toll-like receptor 7; Casp4, caspase 4; Fas, Fas cell surface death receptor; Tlr2, toll-like receptor 2; Tlr9, toll-like receptor 9; Tifab, TIFA inhibitor; Cd68, Cd68 molecule; Msr-1, macrophage scavenger receptor 1; Iba-1, ionized calcium binding adaptor molecule 1; Cd11b, Complement receptor 3; Mrc1, mannose receptor, C type 1; Cd86, CD86 molecule; Tgfβ1, transforming growth factor, beta 1; Arg1, arginase 1; IL4rα, interleukin 4 receptor; Runx3, runt-related transcription factor 3; Pax-1, paired box 1; Batf3, basic leucine zipper ATF-like transcription factor 3; Sp5, Sp5 transcription factor; Hlx, H2.0-like homeobox; Nfkbiz, NFKB inhibitor zeta; Spi1, Spi-1 proto-oncogene; Fli1, Fli-1 proto-oncogene; Lst1, leukocyte specific transcript 1; Maff, MAF bZIP transcription factor F; Elf4, E74 like ETS transcription factor 4; Vgl13, vestigial-like family member 3; Nr2f2, nuclear receptor subfamily 2; Cartpt, CART prepropeptide; Six3, SIX homeobox 3; Meox1, mesenchyme homeobox 1; Tfap-2c, transcription factor AP-2 gamma; Ebf3, EBF transcription factor 3; Mkx, mohawk homeobox; Mei4, meiotic double-stranded break formation protein 4)*.

In addition, cytokine signaling-3 (SOCS_3_) and TLR gene transcripts were upregulated in the hippocampus, and minocycline suppressed the upregulation of SOCSs and Tlr8, Tlr1, Tlr13, Tlr7, Tlr2, and Tlr9 gene transcripts in CCI rats. We found that, after nerve injury, Tlr4 transcripts were only upregulated by <1 fold (Tlr4: fold = 0.56, FDR = 0.0167). The Tnf-α and Nlrp3 transcripts were upregulated by >1 fold (Tnf-α: fold = 1.492, FDR = 0.037; Nlrp3: fold = 1.21, FDR = 4.18E-23) in the hippocampus of CCI rats. Moreover, the upregulated Tnf-α and Nlrp3 gene transcripts were only moderately suppressed by minocycline (Tnf-α: fold = 0.469, FDR = 0.275; Nlrp3: fold = 0.79, FDR = 5.55E-12). Besides, Nlrp1a gene transcripts were only slightly upregulated (Nlrp1a: fold = 0.29, FDR = 0.007). For these reasons, the changes of Tnf-α, Tlr4, and Nlrp gene transcripts are not listed in Table 3. At last, we noted that C3, Ptges, Mt1, Il20rb, Il21r, Il2rb, Hpgds, Il1r1, Tlr8, Card11, P2ry6, Casp4, Fas, and Tifab were upregulated in the CCI group as compared with sham rats. Afterward, the upregulation of these gene transcripts was almost diminished after repeated treatment with minocycline.

More studies have suggested that Cd68, Iba-1 (ionized calcium-binding adaptor molecule-1, involved in microglial motility), Ox-42 (Cd11b, involved in microglial plasticity and motility), Msr-1(macrophage scavenger receptor 1, involved in phagocytosis), and Mhc-II (major histocompatibility complex II) are common markers of microglia activation (Booth and Thomas, 1991; Minett et al., 2016). As shown in Table 3, we found that, compared with sham rats, the Cd68, Msr-1, and Iba-1 transcripts were upregulated by >1 fold in hippocampus of CCI rats. After repeated treatment with minocycline, the Cd68, Msr-1, and Iba-1 transcripts were all obviously downregulated by >1 fold as compared with CCI rats. To our surprise, the Cd11b transcripts were upregulated by <1 fold (fold = 0.72, FDR = 9.34E-30) as compared with sham rats. Moreover, after minocycline treatment, the Cd11b transcripts were only downregulated by <1 fold (fold = 0.55, FDR = 7.70E-19) as compared with CCI rats.

It is clear that microglia/macrophages respond to acute brain injury by becoming activated and developing a pro-inflammatory profile of M1-like or anti-inflammatory profile of M2-like phenotypes (Perego et al., 2013; Luo et al., 2018). According to previous studies, M1 polarization could be determined by the expression levels of Cd86, as well as Il1β, Ccl2, Ccl3, and iNOS. M2 polarization could be ascertained by the increased expression of Arg1, Tgfβ1 (transforming growth factor beta 1), and Il4rα (Pusic et al., 2014; Wu et al., 2014; Ji et al., 2018; Luo et al., 2018). Cd206 (mannose receptor 1, Mrc1) is present in M1 and M2a microglia (Pusic et al., 2014; Wu et al., 2014; Ji et al., 2018; Luo et al., 2018). We noticed that peripheral nerve injury increased the expression of Cd86, Il1β, iNos, Ptges, Ccl2, Ccl3, and Mrc-1. At the same time, we also observed the increased expression of Tgfβ1, Il4rα, and Socs3. However, the expression of Arg1, another M2 marker, is decreased. It appears that minocycline obviously inhibits M1 activation (decreased expression of Cd86, Il1β, iNos, Ptges, and Mrc-1), thus reducing production of cytokines including Il1β, NO, PGs, Ccl2, and Ccl3 in CCI rats. On the other hand, as shown in Table 3, we found that, compared with sham rats, the Tgfβ1 and Il4rα transcripts were upregulated >1 fold (Tgfβ1: fold = 1.16, FDR = 2.58 E-49; Il4rα: fold = 1.03, FDR = 5.18 E-70) in the hippocampus of CCI rats. After repeated treatment with minocycline, the Tgfβ1 and IL4rα transcripts were downregulated by <1 fold as compared with CCI rats. Compared with sham rats, the Arg1 transcripts were downregulated by >1 fold in the hippocampus of CCI rats. Treatment with minocycline only slightly upregulated the transcriptional level of Arg1.

It is well known that some transcription factors have been shown to be directly or indirectly associated with the expression of inflammation-related cytokine genes. Compared with the sham group, the upregulated transcription factor genes were maff, Elf4, Nr2f2, Vgl13, Lst1, Runx3, Tfec, Sp5, Nfkbiz, Hlx, Spi1 (Pu.1), Fli1, Batf3, and Pax-1. Among them, we would like to mention that the levels of gene transcripts of Runx3, Tfec, Sp5, Nfkbiz, Hlx, Spi1, Fli1, Batf3, and Pax-1 were largely suppressed by minocycline. It is noteworthy that gene transcripts of Tfec, Nfkbiz, Hlx, Spil, Fli1, and Batf3 and Pax-1 were obviously increased in the CCI group compared with those of sham rats, and the expression of these genes returned to normal level after minocycline administration. On the other hand, as shown in Table 3, we found that, compared with sham rats, the Maff, Elf4, Vgl13, and Nr2f2 transcripts were upregulated by >1 fold in the hippocampus of CCI rats. After repeated treatment with minocycline, the Maff, Elf4, Vgl13, and Nr2f2 transcripts were slightly downregulated by <1 fold as compared with CCI rats. In addition, compared with sham rats, the Cartpt, Six3, Meox1, Tfap-2c, Ebf3, Mkx, and Mei4 transcripts were downregulated by >1 fold in the hippocampus of CCI rats. Among these transcripts, the Meox1 were obviously reversed by minocycline by >1 fold in the hippocampus of minocycline-treated CCI rats. However, the Cartpt, Six3, Tfap-2c, Ebf3, mkx, and Mei4 transcripts were not obviously reversed by <1 fold in minocycline-treated CCI rats. Individual genes in each category are listed below:

1. only upregulated in CCI rats: Maff, Elf4, Nr2f2, Vgl13, Lst1;
2. only downregulated in CCI rats: Cartpt, Six3, Ap-2c, Ebf3, Mei4;
3. upregulated in CCI and downregulated by minocycline: Runx3, Tfec, Sp5, Nfkbiz, Hlx, Spi1 (Pu.1), Fli1, Batf3, Pax-1; and
4. downregulated in CCI and upregulated by minocycline: Meox1.

### Validation of Microarray Results

**Figure 4:**
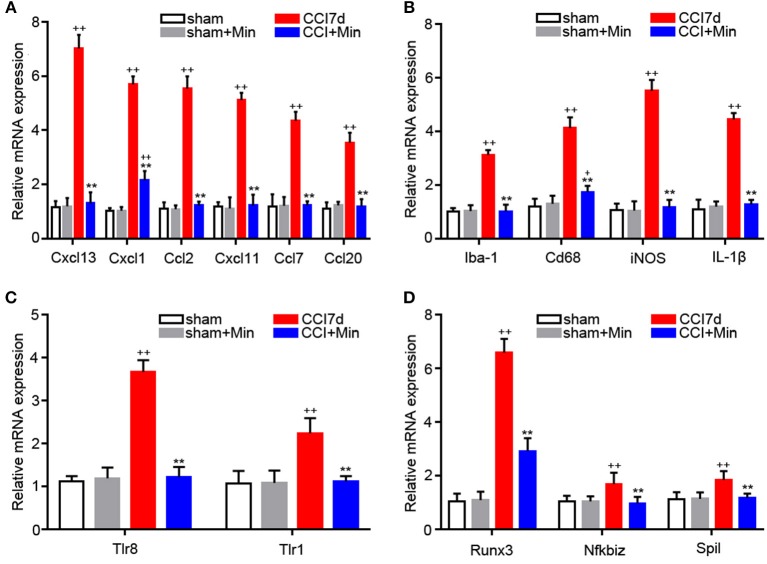
RT-PCR showing the expression of Cxcl13, Cxcl1, Ccl2, Cxcl11, Ccl7, Ccl20, Iba-1, CD68, iNOS, IL-1β, TLR8, TLR1, Runx3, Nfkbiz, and Spil mRNA in the rat hippocampus (*n* = 6). ^+^*P* < 0.05 and ^++^*P* < 0.01, compared with both the sham and sham+Minociclyne groups; ^**^P < 0.01, compared with the CCI 7d group; this applies for all the genes. **(A)** The expression of CXCL13, CXCL1, CCL2, CXCL11, CCL7, CCL20 in the hippocampus. **(B)** The expression of Iba-1, CD68, iNOS, and IL-1β in the hippocampus. **(C)** The expression of TLR8 and TLR1 in the hippocampus. **(D)** The expression of Runx3, Nfkbiz, and Spil in the hippocampus.

Many of the genes that were identified by microarray analysis should be subject to validation by RT-PCR. As shown in Figure 4, we observed that there has been a tacit agreement between the microarray and the PCR gene expression data in terms of changes in both magnitude and direction. The PCR data show that CCI induced the increased expression of cytokines (CXCL13, CXCL1, CCL2, CXCL11, CCL7, and CCL20), TLRs (TLR8 and TLR1), Iba-1 and M1 polarization markers (Cd68, iNOS, IL-1β), and transcription factors (Runx3, Nfkbiz, and Spil). The administration of minocycline did not change the expression of these inflammation-related genes in sham-operated rats (Figure 4). In agreement with the microarray data, minocycline treatment obviously suppressed the elevation in mRNA levels of these genes. Minocycline significantly diminished the upregulated Cd68, iNOS, and Il1β. It appears that transcription factors Runx3, Nfkbiz, and Spil may be involved in the minocycline-mediated analgesic effect and the increased production of inflammation-related cytokines in the hippocampus of neuropathic pain rats. Finally, we found that, between sham and minocycline-treated CCI rats, the expression of the inflammatory-related cytokines (Cxcl13, Ccl2, Cxcl11, Ccl7, and Ccl20), TLRs (Tlr8 and Tlr1), Iba-1 and M1 polarization markers (Cd68, iNOS, and Il1β), and transcription factor (Nfkbiz and Spil) have no statistical significance (Figure 4), which imply that, after minocycline treatment, the upregulated gene expression in CCI rats has returned to normal. On the other hand, between sham and minocycline-treated CCI rats, the expression of Cxcl1 and Runx3 was only partly suppressed by minocycline, which implies that these two genes' expression may be only partly modulated by microglia activity.

## Discussion

We reported here the hippocampal genome-wide transcriptome profiling of rats in neuropathic pain status to elucidate minocycline-mediated analgesic effect at the molecular level. It is well known that the CCI model of neuropathic pain displays some symptoms that are very common in neuropathic pain patients including mechanical and thermal allodynia. Then, we screened the hippocampus of the CCI rats for DEGs.

It has also been proved that minocycline exerts an anti-nociceptive effect in different pain models. Recent studies revealed that the hippocampal CA1 region is more sensitive to ischemic injury and peripheral inflammatory stimulation (Sun et al., 2016; Song et al., 2018). In the present study, CCI operation reduced the threshold of paw withdrawal to a mechanical stimulation. After minocycline treatment, this mechanical allodynia was progressively reduced from 1 to 7 days, which suggests that minocycline reduced pain hypersensitivity by modulating the microglia function within the hippocampus at the early stage of neuropathic pain. On the other hand, it was reported that the onset of depressive-like behavior in CCI animals was 2 weeks following peripheral nerve injury (Xie et al., 2017; Gong et al., 2018). Moreover, minocycline treatment suppressed hippocampal cytokine accumulation and depression-like behaviors in different animal models of chronic pain, such as posttraumatic stress disorder-pain comorbidity (Sun et al., 2016), visceral pain (Zhang et al., 2016), bone Cancer Pain (Dai et al., 2019), and infant nerve injury (Gong et al., 2018). For this reason, we may propose here that minocycline treatment might reduce the risk of nerve injury-induced depression. More studies should be performed to detect the relationship between depression and chronic pain and the effects of minocycline.

We observed that minocycline at 1 μg/μl induced significant analgesic effect in comparison to CCI rats. Minocycline at doses of 2 and 5 μg/μl showed better analgesic effects in comparison to minocycline at a dose of 1 μg/μl. Minocycline at a dose of 5 μg/μl showed apparent elevations of the MWT in comparison with minocycline at a dose of 2 μg/μl. On the other hand, minocycline at 10 μg/μl produced a moderate antinociceptive effect in CCI rats. Minocycline at 15 μg/μl produced a slight but significant nociceptive effect. It seems the minimum dose of minocycline at 5 μg/μl shows the maximum analgesic effect. Recently, several studies also showed the negative action of minocycline in animal or cellular models for nervous system disorders. Similarly to what we observed in CCI rats, Matsukawa et al. also support the idea that neuroprotection is dose-dependent, in that only low doses of minocycline inhibit neuronal cell death cascades at the acute stroke phase, whereas high doses exacerbate ischemic injury (Matsukawa et al., 2009). A low dose of minocycline (25 mg/kg) showed protective effects, with reduced retinal ganglion cell loss and microglial activation, while a high dose of minocycline (100 mg/kg) showed damage effects, with more retinal ganglion cell loss and microglial activation in mice with retinal ischemia-reperfusion injury (Huang et al., 2018). An *in vivo* experiment from Li et al. also showed that intraperitoneal minocycline treatment (45 mg/kg) may induce delayed activation of microglia in aged rats and thus cannot prevent postoperative cognitive dysfunction (Li et al., 2018). For this reason, although our results do not directly investigate the influence and relevant mechanism of high doses of minocycline (10 and 15 μg/μl) on the neuronal excitability or synaptic strength, the present study suggests the possibility that a high dose of minocycline might regulate cell function in neuronal or non-neuronal cells within the hippocampus of CCI rats. In our experiments, the molecular weight of minocycline hydrochloride was 493.94. Then, 494 μg/μl corresponds to 1 M and 5 μg/μl to 10^4^ μM, 10 μg/μl corresponds to 2 × 10^4^ μM and 15 μg/μl corresponds to 3 × 10^4^ μM. Pinkernelle et al. reported that application of 10 μM minocycline (24 h) was deleterious for spinal motor neuron survival (Pinkernelle et al., 2013). Incubation with 50 μM minocycline (24 h) resulted in increased cell metabolic activity in primary glial cultures. Application of 100 μM minocycline inhibited astroglia migration (24 h) and upregulated the elevated Cx43 protein expression (72 h) in rat spinal cord slices (Pinkernelle et al., 2013). A high dose of minocycline attenuated reductions in O1- and O4-positive oligodendrocyte progenitor cells and myelin content in hypoxia-ischemia-induced neuroinflammation and white matter injury in rats (Carty et al., 2008). However, we also noticed that 10 and 15 μg/μl showed poor analgesic effects within 2 h after minocycline treatment. A study on the protective effect of minocycline on ischemic stroke from Matsukawa et al. indicated that 75 min incubation with 10 μM minocycline induced increased Bcl-2 protein expression in striatum neurons. Moreover, application of 10 or 100 μM minocycline for 4 h displayed toxicity to both neurons and astrocytes in the striatum (Matsukawa et al., 2009). We also noticed that minocycline (30 min, 1 h, and 2 h after its injection) at doses of 10 and 15 μg/μl showed poor analgesic effects in comparison with minocycline at a dose of 5 μg/μl, and the poor effect was sustained for 7 days. It seems that the adverse effects of minocycline on neurons or non-neuronal cells may have occurred in a short period of time. Of course, the influence of different doses of minocycline on neurons or non-neuronal cells in the hippocampus remains to be further studied.

We found that, in the sham group vs. CCI group and minocycline-treated group vs. CCI group, the top 2 items of KEGG pathway are cytokine-cytokine receptor interaction and TLR pathway, which indicates that minocycline administration can regulate the expression of genes in these two pathways, and reversing these gene expression changes may be considered as one of the important reasons for minocycline-mediated analgesic effect. Nerve damage leads to glial activation and thus facilitates the production and release of pronociceptive factors such as interleukins and chemokines from glial cells. We noticed that, after sciatic nerve injury, IL-1β was the most striking interleukin that increased most seriously in hippocampus of CCI rats. Moreover, the increased gene expression of CXCL13, CXCL1, CCL2, CXCL11, and CCL7 in the rat hippocampus was observed after nerve damage. The increased chemokine expression was obviously suppressed by intra-hippocampal injection of minocycline. It appears that minocycline was able to reduce microglia activity efficiently, which led to the decreased expression of these genes. In addition, the increased expression of interleukins and chemokines should be regulated by some transcription factors. For example, the elevated expression of IL-1β was associated with binding of transcription factor Spil/Pu.1 to IL-1β promoter in activated inflammatory macrophage (Vanoni et al., 2017). Spil/Pu.1 can also bind to the CCL2 promoter and stimulate its expression (Sarma et al., 2014). Runx3 knockdown can induce the downregulation of CXCL11 in lung cancer cells (Kim et al., 2015). IκBz can function as a transcriptional activator of CXCL1 and CCL2, which are involved in inflammatory responses (Hildebrand et al., 2013; Brennenstuhl et al., 2015). Similarly, we also found that, compared to sham rats, IL-1β, CXCL1, CXCL11, CCL2, and transcription factor (Spil/Pu.1, Runx3, and IκBz) are obviously elevated at 7 days following nerve injury. After treatment with minocycline, these interleukins and chemokines and transcription factor were obviously decreased. It seems that the increased expression of interleukins and chemokines may be regulated by these transcription factors in the rat hippocampus after nerve injury.

In addition, nerve injury evoked the elevated expression of many different kinds of TLRs (TLR8, TLR1, TLR13, TLR7, TLR2, and TLR9) in the rat hippocampus. After treatment with minocycline, the elevated expression of these TLRs in the hippocampus was significantly lower compared to the CCI group. More recent studies suggest that TLRs play an important role in immune response by producing inflammatory cytokines and chemokines under pathological conditions. For example, TLR1, TLR2, TLR7, and TLR9 activation stimulated the production of IL-1β and MCP-1 in B cells (Agrawal and Gupta, 2011). TLR2 activation led to the accumulation of IL-1β and chemokines (CCL7, CCL8, CCL9, CXCL1, CXCL2, CXCL4, and CXCL5) in primary mouse microglial cells (Aravalli et al., 2005). TLR7 and TLR9 stimulation led to the accumulation of IL-1β, CCL2, CCL3, CXCL1, CXCL9, and CXCL10 in mouse brain (Butchi et al., 2011). It is reasonable to speculate that, in the hippocampus of CCI rats, activation of TLR signaling in the hippocampus by peripheral nerve injury may partially participate in the increased expression of these inflammatory cytokines or chemokines.

Some previous studies demonstrate that IκBz can serve as a nuclear inhibitor of NF-κB and is thought to have a key role in inflammatory responses. On the other hand, IκBz is induced quickly in monocytes and macrophages after LPS stimulation (Yamazaki et al., 2001). In the present experiments, the CCI-induced increased expression of IκBz was completely impaired in minocycline-treated CCI rats, suggesting a role for microglia activation in upregulated IκBz expression. It was reported that IκBz is obviously induced in macrophages after TLR or IL-1R stimulation (Hanihara et al., 2013). In chronic lymphocytic leukemia cells, TLR9 activation can lead to the increased IκBz expression and IgM release (Fonte et al., 2017). Inhibition of TLR1/TLR2 signaling suppressed D39-evoked IκBz expression in human monocyte (Sundaram et al., 2016). On the other hand, promoting IκBz degradation inhibits TLR-mediated inflammation and disorders (Hanihara-Tatsuzawa et al., 2014). Similarly, the absence of IκBz obviously suppressed B-cell activation and proliferation after TLR activation (Kimura et al., 2018). We also noted that TLR8, TLR1, TLR13, TLR7, TLR2, TLR9, and IκBz gene expression robustly increased in the hippocampus, and the expression was obviously impaired in minocycline-treated CCI rats. One possible explanation is that the upregulated IκBz gene expression may be associated with the increased TLR expression. In addition, microglia-mediated inflammatory reaction plays a double role in some nervous diseases due to two distinct phenotypes, including the neurotoxic reactive phenotype (M1) and neuroprotective M2 (Kobayashi et al., 2013; Tang and Le, 2016). In the present study, minocycline inhibits M1 activation, thus leading to decreased expression of inflammatory factors including IL-1β, CCL2, CCL3, and iNOS. Thus, it can be seen that dampening of M1 polarization is another possible mechanism of minocycline-medicated analgesia.

In summary, the DEGs were identified, and many inflammation-related genes including TLRs and chemokines were considered as important genes in the formation of neuropathic pain through pathway analysis of microarray data, which may help us to further understand the underlying molecular mechanisms of chronic pain. After the bioinformatics analysis of gene expression profiles, the expression of inflammation-related genes was further identified via the RT-PCR method. Although the results obtained from our experiments indicate that intra-hippocampal injection of minocycline exerts an analgesic effect and many inflammation-related genes may be involved in the formation of neuropathic pain, the study we conducted also has certain limitations that should be considered in future studies. In other words, further studies are required to further explore the roles of these inflammation-related genes in the hippocampus, where it is implicated in the formation of the neuropathic pain.

## Data Availability Statement

Publicly available datasets were analyzed in this study. This data can be found here: https://pan.baidu.com/s/1dZ4ImpqLIEqWkF3gN2llDw.

## Ethics Statement

The protocol was prepared from SD rats in accordance with the National Institutes of Health guidelines in a manner that minimized animal suffering and animal numbers. All experiments were carried out in accordance with China animal welfare legislation and were approved by the Zunyi Medical University Committee on Ethics in the Care and Use of Laboratory Animals.

## Author Contributions

JZ, YC, and XL: conceived and designed the experiments. LH, RX, and HT: animal experiment. LH and TX: behavioral assessment of pain. LH, RX, YP, and SC: analyzed the data. JZ, LH, and XL: wrote the paper.

### Conflict of Interest

The authors declare that the research was conducted in the absence of any commercial or financial relationships that could be construed as a potential conflict of interest.
